# The *Bifidobacterium adolescentis* BAD_1527 gene encodes GH43_22 α-L-arabinofuranosidase of AXH-m type

**DOI:** 10.1186/s13568-024-01738-9

**Published:** 2024-07-20

**Authors:** Walid Fathallah, Vladimír Puchart

**Affiliations:** 1 Institute of Chemistry, Slovak Academy of Sciences, Dúbravská cesta 9, 845 38 Bratislava, Slovakia; 2 Faculty of Science, Beni-Suef University, Beni-Suef, 625 11 Egypt

**Keywords:** *Bifidobacterium adolescentis*, α-L-Arabinofuranosidase, Arabinoxylan, Arabinan, Substrate specificity, Positional specificity

## Abstract

*Bifidobacterium adolescentis* gene BAD_1527 has previously been suggested to code for a β-xylosidase (Kobayashi et al., Mar Drugs 18:174, 2020). Our detailed investigation of the substrate specificity of the GH43_22 protein using a wide spectrum of natural and artificial substrates showed that the enzyme hydrolyzed neither linear xylooligosaccharides nor glucuronoxylan. Xylose was released only from the artificial 4-nitrophenyl β-D-xylopyranoside (1.58 mU/mg). The corresponding α-L-arabinofuranoside was by three orders of magnitude better substrate (2.17 U/mg). Arabinose was the only monosaccharide liberated from arabinoxylan and α-1,3- or α-1,2-singly arabinosylated xylooligosaccharides. Moreover, the enzyme efficiently debranched sugar beet arabinan and singly arabinosylated α-1,5-L-arabinooligosaccharides, although short linear α-1,5-L-arabinooligosaccharides were also slowly degraded. On the other hand, debranched arabinan, arabinogalactan as well as 2,3-doubly arabinosylated main chain residues of arabinan and arabinoxylan did not serve as substrates. Thus, the enzyme encoded by the BAD_1527 gene is a typical α-L-arabinofuranosidase of AXH-m specificity.

**Supplementary Information:**

The online version contains supplementary material available at 10.1186/s13568-024-01738-9.

## Key points

- BAD_1527 gene encodes a protein releasing xylose from NPX, but not natural substrates
- L-Arabinose is released from natural substrates, similarly to other GH43_22 members
- The enzyme debranches arabinoxylan, branched arabinan and derived oligosaccharides

**Supplementary Information:**

The online version contains supplementary material available at 10.1186/s13568-024-01738-9.

## Introduction

Bifidobacteria are Gram-positive, anaerobic health-promoting probiotic bacteria found in the human gut, playing a pivotal role in the health of human beings (Wang and Zhong 2024). In adults, *Bifidobacterium adolescentis* is one of the predominant bifidobacterial species inhabiting the intestines (Derrien et al. 2022). The genomic analysis of *B. adolescentis* revealed the presence of genes encoding carbohydrate-active enzymes (Duranti et al. 2016) hydrolyzing recalcitrant polysaccharides and derived oligosaccharides that highly impact the growth of this probiotic species and are involved in the production of valuable metabolites (Rudjito et al. 2023).

Arabinose is one of the most abundant pentoses in nature and a common component of several plant hemicellulosic (arabinoxylan; AX) and pectic polysaccharides (arabinan and arabinogalactan (AG)). Arabinoxylan is a heteropolymer having a main chain composed of β-1,4-linked d-xylopyranosyl (Xyl*p*) residues that may be singly or doubly branched with α-L-arabinofuranosyl (Ara*f*) groups at position 2 and/or 3 (Liu et al. 2021), while arabinan is a homopolymer having a backbone of α-1,5-linked Ara*f* units, which can be substituted with Ara*f* residues at *O*-3 and/or *O*-2 position. If these Ara*f* side chains are enzymatically cleaved off, the resulting linear polysaccharide is called debranched arabinan (DA) (Park et al. 2015). The molecular diversity of natural arabinose-containing polysaccharides implies the requirement of multiple enzymes for their complete deconstruction, including endo-1,5-α-L-arabinanases (EC 3.2.1.99), α-L-arabinofuranosidases (E.C 3.2.1.55), β-xylosidases (EC 3.2.1.37) and endo-1,4-β-D-xylanases (EC 3.2.1.8) that often act synergistically. Furthermore, some glycosidases display dual or multiple functions due to the conformational similarity of potential substrates, e.g., β-D-xylopyranose and α-L-arabinofuranose, resulting in substrate promiscuity (Zhang et al. 2021).

α-L-Arabinofuranosidases are exo-acting glycoside hydrolases that cleave α-1,2-, α-1,3- or α-1,5-arabinofuranosidic linkages to release the non-reducing-end terminal Ara*f* residues from plant polysaccharides such as AX, arabinan, AG, and the corresponding oligosaccharides derived thereof (Taylor et al. 2006). However, the α-L-arabinofuranosidases may display variable substrate and positional preference, therefore, the enzymes hydrolyzing polymeric AX are designated arabinoxylan arabinofuranohydrolases (AXHs) (Kormelink et al. 1991). In addition, on the basis of the linkages hydrolyzed, the AXHs are distinguished into two common categories: AXH-m and AXH-d3. The enzymes that liberate Ara*f* solely from 2-*O*- or 3-*O*-monoarabinosylated Xyl*p* residues represent the AXH-m specificity (Kormelink et al. 1993), while AXH-d3 arabinofuranosidases attack 3-*O*-linked Ara*f* substituents exclusively from 2,3-doubly decorated Xyl*p* moieties (Van Laere et al. 1997). According to the CAZy classification (http://www.cazy.org/), a vast majority of the AXHs are classified into glycoside hydrolase families GH43, GH51, GH54 and GH62 (Si et al. 2023). So far, GH43 is one of the largest and most diverse CAZy families, currently being divided into 38 subfamilies. The gene BAD_1527 from *Bifidobacterium adolescentis* ATCC 15703 has been suggested to code for a xylanolytic protein belonging to GH43_22 subfamily, putatively assigned as a β-xylosidase (Kobayashi et al. 2020) due to a limited hydrolysis of xylooligosaccharides (XOS) and 4-nitrophenyl β-D-xylopyranoside. However, its α-L-arabinofuranosidase capacity has never been tested although all other hitherto characterized GH43_22 members have been reported to be α-L-arabinofuranosidases (http://www.cazy.org/GH43_22_characterized.html).

In this work, substrate specificity of the protein encoded by the BAD_1527 gene was investigated in detail. It was revealed that the GH43_22 protein does not hydrolyze linear xylooligosaccharides (Xyl_2-4_) and behaves as a debranching α-L-arabinofuranosidase of the AXH-m specificity exhibiting activity on both arabinoxylan and arabinan and the corresponding singly branched oligosaccharides.

## Materials and methods

### Enzyme and chemicals

Cloning of the *B.* *adolescentis* BAD_1527 gene and its heterologous expression in *Escherichia coli* was performed by NZYTech (Lisbon, Portugal). The expressed protein (GenBank accession: BAF40308.1; UniProt accession: A1A3M5) referred as *Ba*Xyl43A (product code: CZ0389) was purchased from the supplier (NZYTech). The purchased product was used without any further modification (with exception of dilution). It was supplied at 1 mg/mL concentration and its electrophoretic (molecular weight approximately 41.1 kDa according to SDS PAGE) purity was demonstrated by the supplier (https://www.nzytech.com/media/dds/brochurescertificates/cz0389_ug_en_v2302.pdf). 4-Nitrophenyl α-L-arabinofuranoside (NPA) and 4-nitrophenyl β-D-xylopyranoside (NPX) were supplied by Carbosynth (Compton, UK) and a series of natural substrates were procured from Megazyme (Bray, Ireland). These substrates encompassed linear β-1,4-xylooligosaccharides (XOS): (xylobiose Xyl_2_; xylotriose Xyl_3_; and xylotetraose Xyl_4_), arabinoxylooligosaccharides (AXOS) (3^2^-α-L-arabinofuranosyl-xylobiose A^3^X, 2^3^-α-L-arabinofuranosyl-xylotriose A^2^XX, 3^3^-α-L-arabinofuranosyl-xylotetraose XA^3^XX, a mixture of 2^3^-α-L-arabinofuranosyl-xylotetraose XA^2^XX and XA^3^XX, 2^3^,3^3^-di-α-L-arabinofuranosyl-xylotriose A^2,3^XX and 2^3^,3^3^-di-α-L-arabinofuranosyl-xylotetraose XA^2,3^XX), linear α-1,5-L-arabinooligosaccharides (AOS) (arabinobiose Ara_2_, arabinotriose Ara_3_ and arabinotetraose Ara_4_), branched AOS (3^2^-α-L-arabinofuranosyl-arabinotriose AA^3^A and a mixture of 2^2^,3^2^-di-α-L-arabinofuranosyl-arabinotriose AA^2,3^A and 3^2^-α-L-arabinofuranosyl-arabinotetraose AAA^3^A) as well as polysaccharides: wheat arabinoxylan (medium-viscosity; arabinose:xylose = 38:62; WAX), rye arabinoxylan (high-viscosity; arabinose:xylose = 40:60; RAX), debranched arabinan (from sugar beet pulp; arabinose:galactose:rhamnose = 71:26:3; DA), branched arabinan (from sugar beet pulp; arabinose:galactose:rhamnose:galacturonic acid:other sugars = 69:18.7:1.4:10.2:0.7; SBA) and arabinogalactan (from larchwood; galactose:arabinose:other sugars = 81:14:5; AG). In addition, glucuronoxylan (from beechwood; GX) prepared according to Ebringerová et al. (1967) was also used.

### Enzyme assays

All enzyme assays were performed at 35 °C in 50 mM sodium phosphate buffer, pH 6.0. All control experiments were run in parallel in the absence of the enzyme. α-L-Arabinofuranosidase and β-xylosidase activities were quantified using 1 mM chromogenic substrates NPA and NPX, respectively (Lagaert et al. 2010), and enzyme concentration of 4 μg/mL. The reaction was terminated by addition of six volumes of saturated aqueous solution of sodium tetraborate. The released 4-nitrophenol was determined spectrophotometrically at 405 nm and quantified (after subtraction of the chromophore released in enzyme-free controls) using a calibration curve constructed in the same way. One unit of enzyme activity is defined as the enzyme amount which liberates one micromole of 4-nitrophenol in one min.

The enzyme specificity was evaluated on the basis of hydrolysis of WAX, RAX, DA, SBA, AG, GX, AXOS, AOS and XOS. The polysaccharides (1% w/v) or the oligosaccharides (0.1% w/v) were treated with 0.01 mg/mL enzyme (final concentration). At time intervals, 2 μL aliquots were spotted onto the TLC plate. No modification of the substrates was observed in the control mixtures.

### Analysis of hydrolysis products

The hydrolysis of natural substrates was followed by thin-layer chromatography (TLC) on aluminum-coated silica gel plates (Merck, Darmstadt, Germany). The polysaccharide and XOS hydrolyzates were developed once in a solvent system of 1-butanol/ethanol/water (10:8:5, by vol.), while the hydrolyzates of AXOS and AOS were developed twice in a solvent system of chloroform/acetic acid/water (6:7:1, by vol.). The plates were air-dried, immersed into the detection reagent [0.5% orcinol (w/v) in 5% sulfuric acid in ethanol (v/v)], dried and the carbohydrates were visualized by heating at 120 °C and quantified by densitometry (UN-SCAN-IT; Silk Scientific, Orem, UT, USA).

### Analysis of the amino acid sequence

The amino acid sequence of the enzyme was compared with other sequences. In the multiple sequence alignment all GH43 proteins listed in Table ST1 (Supplementary Material) were included along with characterized GH43_22 α-L-arabinofuranosidases (*Rj*Abn43A, *Bl*Abf43A, *Bl*Abf43B and *Bl*Abf43E), *Ba*Abf43C and other characterized *B. adolescentis* α-L-arabinofuranosidases (*Ba*AbfA, *Ba*AbfB and *Ba*AXH-d3). All sequences were analyzed for the presence of a signal sequence using the server of SignalP, version 6.0 (https://services.healthtech.dtu.dk/services/SignalP-6.0/). If the signal sequence was predicted to be present, it was removed from further analysis but amino acid numbering was preserved. Multiple sequence alignment was generated by Clustal Omega server (https://www.ebi.ac.uk/jdispatcher/msa/clustalo).

## Results

### The BAD_1527 gene product and its xylanolytic function

**Fig. 1:**
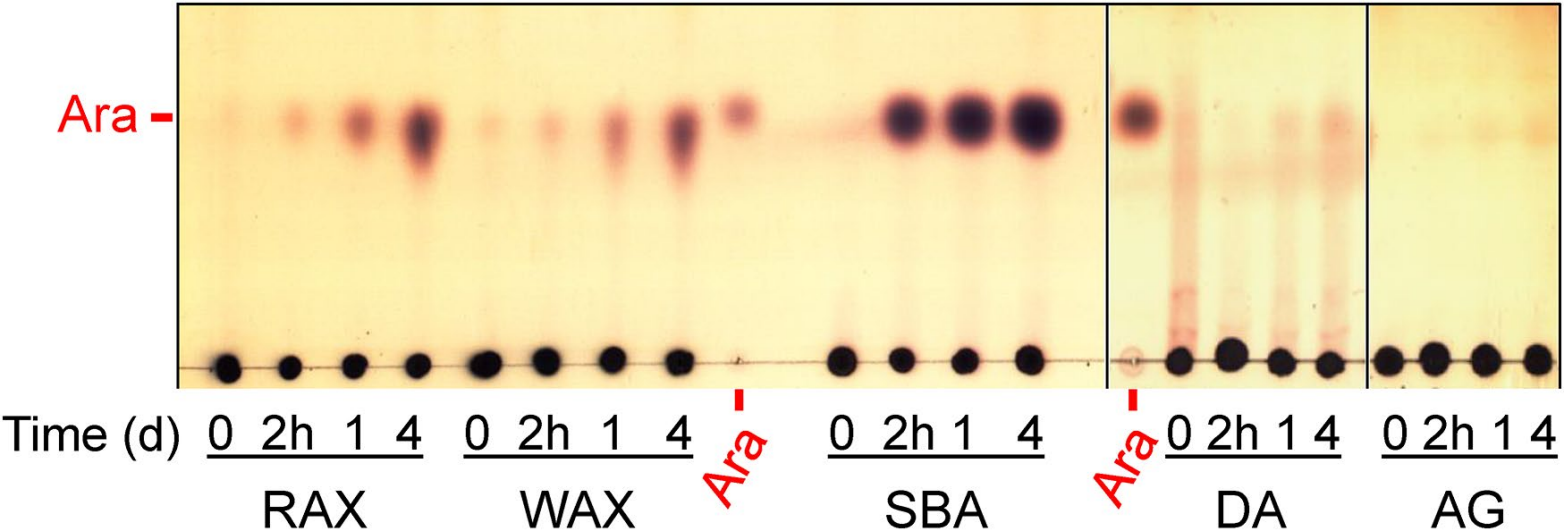
TLC analysis of the products generated from different arabinose-containing polysaccharides: rye arabinoxylan (RAX); wheat arabinoxylan (WAX); branched arabinan (SBA); linear arabinan (DA); and larchwood arabinogalactan (AG) by *Ba*Abf43C from the GH43_22 subfamily. The 1% polysaccharide solutions in 50 mM sodium phosphate buffer, pH 6.0, were treated with 0.01 mg/mL enzyme at 35 °C. The plate was developed in the solvent system of 1-butanol/ethanol/water (10:8:5, by vol.), and the sugars were visualized using orcinol detection reagent

Although the *B.* *adolescentis* enzyme is classified into GH43_22 subfamily and referred as exo-1,4-β-xylosidase *Ba*Xyl43A by the supplier, we found out that the enzyme (used at even 10-times higher concentration) neither hydrolyzed linear XOS (Supplementary Fig. S1) nor released xylose from polysaccharides GX (Supplementary Fig. S1), WAX and RAX (Fig. 1).

### Activity on chromogenic substrates

Hydrolytic activity of the protein was determined on the chromogenic substrates NPX and NPA. *Ba*Xyl43A showed high specific activity on NPA (2.17 U/mg) which is three orders of magnitude higher than its specific activity on NPX (1.58 mU/mg) (Table 1), suggesting that the enzyme is rather α-L-arabinofuranosidase than β-xylosidase. From this point, we will call the enzyme *Ba*Abf43C.

**Table 1:**
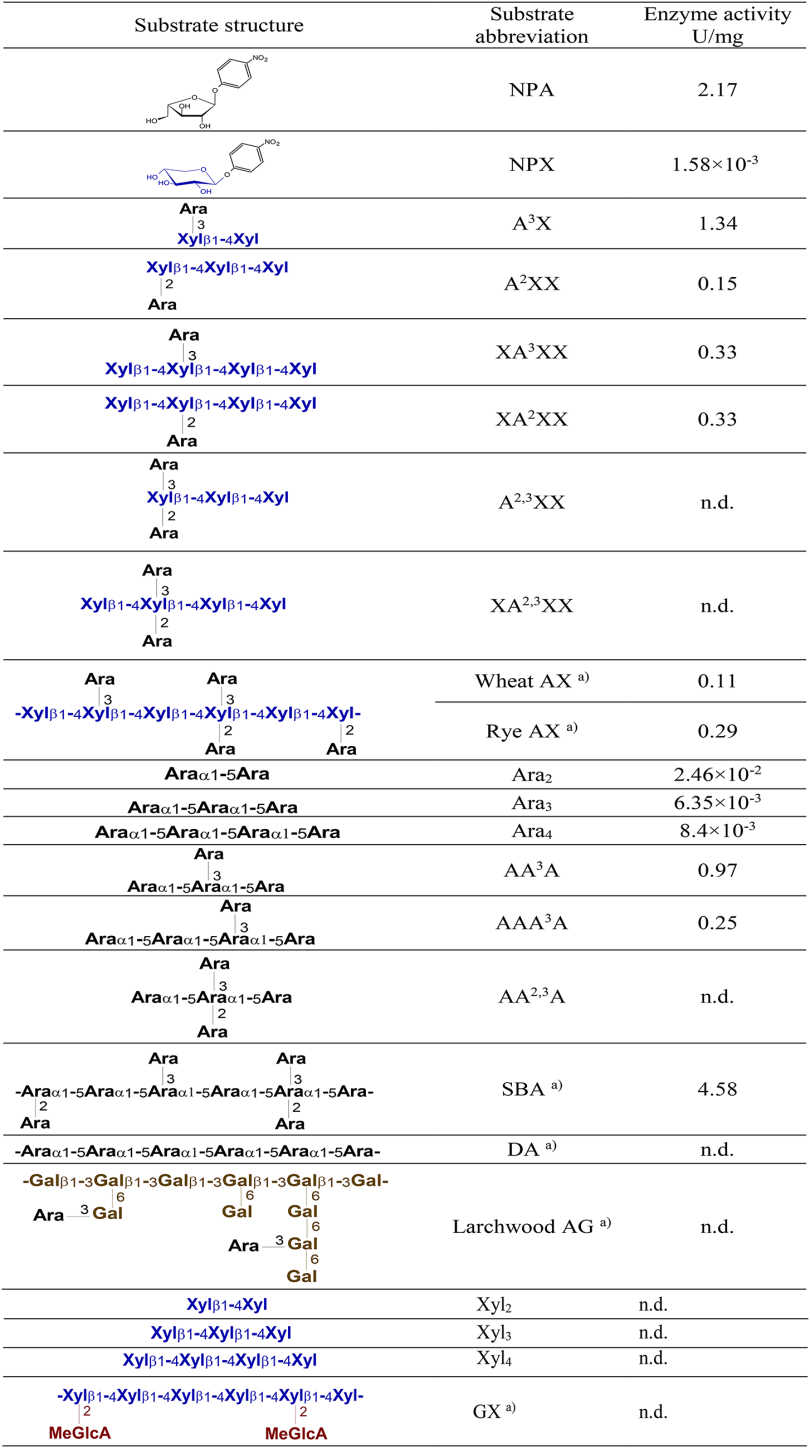
Activity of *Ba*Abf43C against polymeric and oligomeric substrates

*n.d.* not detected

^a^The formulae given in the upmost left column show a simplified structure of the polysaccharides

### Activity on arabinoxylan and arabinoxylooligosaccharides

**Fig. 2:**
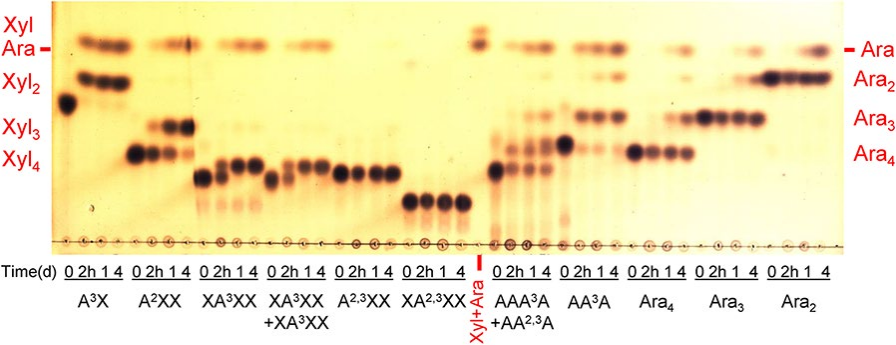
TLC analysis of the products generated from different arabinoxylooligosaccharides and arabinooligosaccharides by *Ba*Abf43C from the GH43_22 subfamily. The 0.1% oligosaccharide solutions in 50 mM sodium phosphate buffer, pH 6.0, were treated with 0.01 mg/mL enzyme at 35 °C. The plate was developed twice in the solvent system of chloroform/acetic acid/water (6:7:1, by vol.), and the sugars were visualized using orcinol detection reagent

The enzyme liberated arabinose from arabinoxylans (WAX and RAX) and the following arabinoxylooligosaccharides: A^3^X, A^2^XX, XA^3^XX and XA^2^XX (Table 1, Figs. 1 and 2). The best substrate was the trisaccharide A^3^X, which was de-arabinosylated within 2 h. Internally substituted substrates XA^3^XX and XA^2^XX were debranched after 1 day regardless of the position of arabinosylation. Interestingly, A^2^XX, which is 2-decorated at the non-reducing end, served as the worst substrate that was not consumed even after 1 day. It is noteworthy that the de-arabinosylated products were not degraded further to shorter xylooligosaccharides, that is in line with the absence of xylose, confirming the lack of β-xylosidase activity towards linear β-1,4-xylooligosaccharides (Fig. 2). Doubly substituted AXOS (A^2,3^XX and XA^2,3^XX) remained intact (Fig. 2).

### Activity on arabinan and arabinooligosaccharides

The removal of arabinose was also observed from singly decorated main chain residues of arabinans and AOS. In fact, these were even better substrates than AX and AXOS except A^3^X (Table 1). Although the enzyme was highly active on branched arabinan (SBA), linear counterpart DA as well as larchwood arabinogalactan were not hydrolyzed (Fig. 1). Again, the enzyme converted singly decorated AOS (AA^3^A and AAA^3^A) to the corresponding linear products (Ara_3_ and Ara_4_, respectively). In contrast, doubly substituted AOS (AA^2,3^A) was resistant to the enzyme (Fig. 2). During the hydrolysis of AAA^3^A two primary products were observed. In addition to linear Ara_4_ generated via debranching of the substrate, another arabinotetraose tentatively identified as AA^3^A was also formed. Its formation is explained by a trimming of the substrate (AAA^3^A) from the non-reducing end. Such a main-chain degrading activity was also observed on short linear AOS (Ara_2_ to Ara_4_). Although their degradation was detected, this exo-1,5-α-L-arabinofuranosidase activity is significantly lower than the debranching activity and is decreasing with the oligosaccharide chain length (Table 1), thus explaining the absence of arabinose release from polymeric DA.

### Structural comparison of *Ba*Abf43C with other GH43 members

**Fig. 3:**
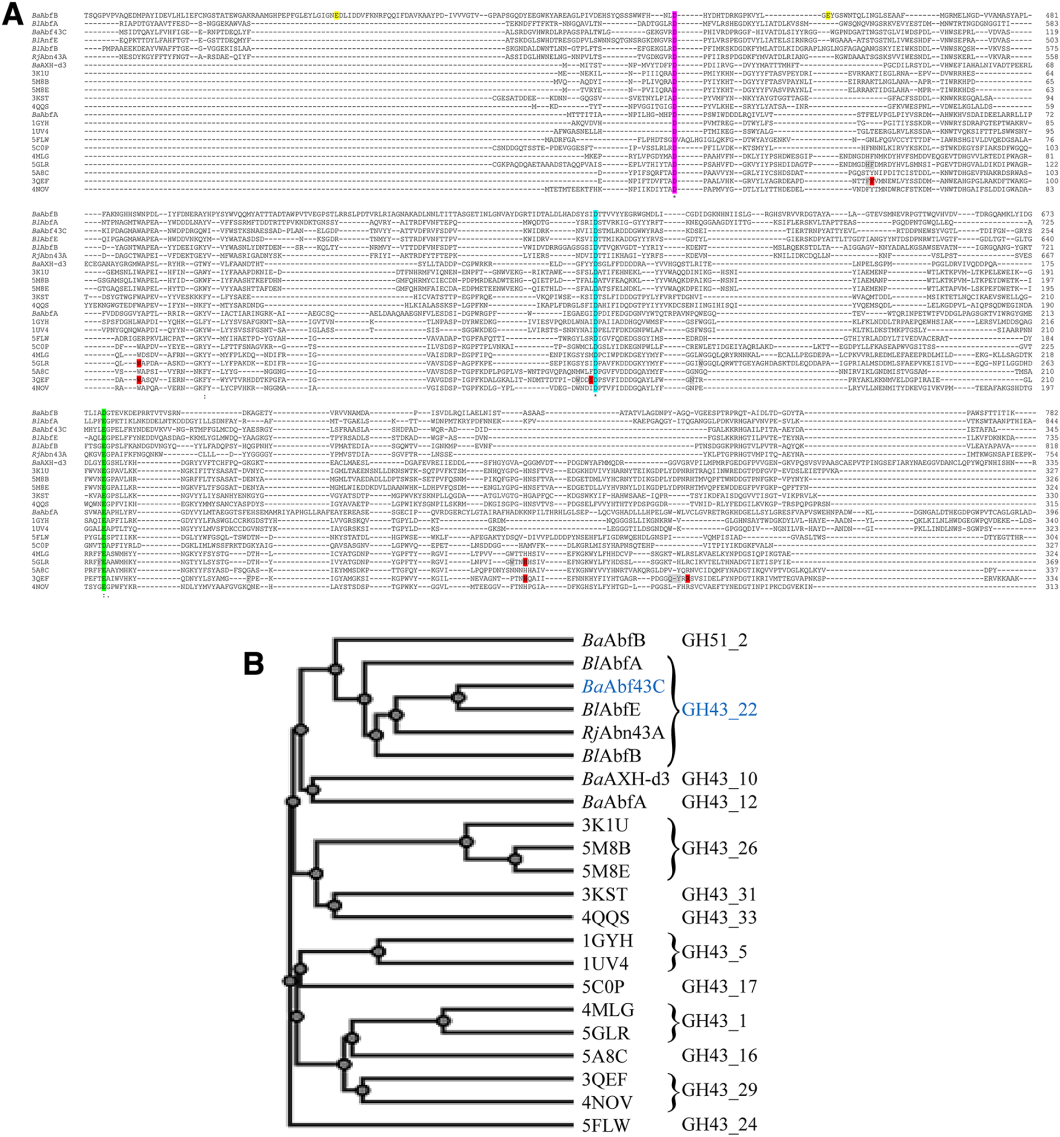
Comparison of *Ba*Abf43C amino acid sequence with other *B.* *adolescentis* α-L-arabinofuranosidases, other characterized GH43_22 members and with GH43 representatives having solved 3-D structure (designated with their PDB codes and better described in Supplementary Material, Table ST1) that showed structural similarity with *Ba*Abf43C model. Panel **A**, multiple sequence alignment of the sequences. Catalytically competent amino acids of the GH43 enzymes, i.e. catalytic base, modulator of catalytic acid, and catalytic acid are violet-, cyan- and green-highlighted, respectively. Catalytic competent amino acids of the GH51 *Ba*AbfB, i.e. catalytic acid/base and catalytic nucleophile, are yellow-highlighted. Amino acids forming the -1 subsite of *Cj*Abf43A (3QEF) and *Co*Xyl43 (5GLR) are shown in red, while amino acids interacting with the oligosaccharide ligands and forming other active site subsites are shown in gray. Panel **B**, phylogenetic tree corresponding to the multiple sequence alignment. Classification of the enzymes into the subfamilies is indicated

Quite surprisingly, only three acidic amino acid residues were found to be conserved in all GH43 sequences analyzed (Fig. 3A). On the basis or their functional identification in other GH43 structures they correspond to catalytic base (D58; *Ba*Abf43C numbering), modulator of catalytic acid (D194) and catalytic acid (E259) (violet, cyan and green highlighted, respectively). Even more interesting is the fact that these aspartates/glutamates are conserved also in GH51 member *Ba*AbfB, although their conservation may not be important, at least from a functional point of view, because this GH51 retaining member adopts a completely different fold [(β/α)_8_-barrel] including distinct catalytic amino acids (yellow highlighted in Fig. 3A). Nevertheless, the multiple sequence alignment and simultaneously generated phylogenetic tree show a relationship between the GH43 enzymes and their division into subfamilies, one of which comprises GH43_22 members including *Ba*Abf43C (Fig. 3B). Although very limited number of the sequences was included in the multiple sequence alignment, the tree is in agreement with a previously published subdivision of the GH43 family (Jones et al. 2018) in a view that subfamilies GH43_10 and GH43_12 are the closest and subfamilies GH43_1, GH43_16, GH43_29 and GH43_24 the most distant to the GH43_22 subfamily.

Unfortunately, the 3-D structure of any GH43_22 representative has not been solved. For structural purposes we used a model of *Ba*Abf43C deposited in AlphaFold database, which has a high confidence. The model predicts that the enzyme structure is so called 5-bladed β-propeller, similar to other GH43 members. Accordingly, upon superposition overall fold of *Ba*Abf43C matches very well the other inverting GH43 enzymes (Supplementary Fig. S2). With the aim to identify amino acids involved in substrate recognition we analyzed structural model of *Ba*Abf43C and the most similar GH43 enzyme complexes having a carbohydrate ligand (Table ST1). These were *Cj*Abf43A complex with AA^3^A (PDB Id: 3QEF) and *Co*Xyl43 complex with xylotriose and arabinose (PDB Id: 5GLR) (Supplementary Material). *Cj*Abf43A is a GH43_29 arabinofuranosidase specifically releasing α-1,2-linked Ara*f* side chain residues from branched arabinan and AOS including 2,3-diarabinosylated substrates. AA^3^A in the complex with *Cj*Abf43A thus represents the product released by the enzyme from AA^2,3^A. The ligand occupies the active site, while subsite -1 (recognizing the cleaved off 2-linked Ara*f* moiety) houses a carbohydrate-mimicking ligand ethylene glycol. In the active site, the following amino acids (gray highlighted in Fig. 3A) have been found to interact with the ligands: F66, F67, W103, W164, I167, W185, F234, H267, Q292, Y293 and R295 (the residues comprising -1 subsite are underlined and they are red highlighted in Fig. 3A), in addition to the catalytic amino acids D41, D168 and E215. Of them, only an equivalent of W103 (W125 in *Co*Xyl43) providing a hydrophobic platform for stacking interaction with the sugar ring bound to– 1 subsite is found in the GH43_22 enzymes (W128 in the *Ba*Abf43C; Fig. 3A, Supplementary Fig. S2). Similar conclusion can be drawn for the residues found in the substrate-binding pocket of GH43_1 β-xylosidase *Co*Xyl43. A comparison of the *Ba*Abf43C model with the structures of *Cj*Abf43A and *Co*Xyl43 revealed that the overall structure is similar, however, the arrangement of the loops around the catalytic site may be different (Supplementary Fig. S2). A detailed analysis of *Ba*Abf43C model showed that its side chain of Y288 would sterically clash with the ligand of the complex *Cj*Abf43A-AA^3^A (Supplementary Figure S2), indicating a different binding of the oligosaccharide, which serves as a substrate for *Ba*Abf43C (Fig. 2). All these data suggest that despite the conservation of the catalytic machinery and overall 3-D structure, the accommodation of the substrates differs between GH43 subfamilies and it is difficult to be estimated without solved 3-D structure of a given GH43_22 representative.

## Discussion

Amaretti et al. (2013) have demonstrated the ability of *B. adolescentis* to degrade linear β-1,4-xylooligosaccharides to xylose. The corresponding β-xylosidase activity has been ascribed to a product encoded by the gene BAD_1527, however, the assignment has been done solely on the basis of the molecular weight of the purified β-xylosidase that was similar to the calculated molecular weight of the BAD_1527 protein product. The heterologous protein produced via expression of the BAD_1527 gene has been later used for hydrolysis of short xylooligosaccharides (Kobayashi et al. 2020). It was reported that the BAD_1527 gene product exhibits relatively low activity on NPX (10 mU/mg at 18.4 mM substrate concentration). This data is in a very good agreement with our observation of specific activity (1.58 mU/mg on 1 mM NPX).

Although Kobayashi et al. (2020) reported extremely low (below 10%) conversion of 10 mM xylotriose and xylobiose, we did not detect xylose release from 2–3 mM linear XOS, even after several days. Using the same substrate and tenfold lower enzyme concentrations, however, we observed time-dependent arabinose liberation from a variety of polymeric and oligomeric substrates. We thus demonstrated that the BAD_1527 gene encodes not β-xylosidase, but in fact α-L-arabinofuranosidase. Therefore, we suggest renaming the corresponding protein to *Ba*Abf43C because it is the third characterized α-L-arabinofuranosidase of *B. adolescentis* from GH43 family, as discussed below. The enzyme is able to release arabinose side chains from arabinoxylan and in particular branched arabinan, but only from singly decorated main chain residues. Accordingly, debranched (linear) arabinan remains intact towards the enzyme treatment, although the enzyme slowly hydrolyzes short α-1,5-L-arabinooligosaccharides. Therefore, the enzyme behaves as a typical AXH-m arabinofuranosidase. Moreover, it releases much more arabinose from the branched arabinan than other α-L-arabinofuranosidases produced by *B.* *adolescentis* (Lagaert et al. 2010; Van Laere et al. 1999), and *Ba*Abf43C is the first *B.* *adolescentis* GH43 α-L-arabinofuranosidase investigated to hydrolyze linear and branched AOS.

The detailed examination of the BAD_1527 gene product broadens a spectrum of α-L-arabinofuranosidases produced by *B.* *adolescentis*. So far, three different arabinose-releasing enzymes have been characterized in the health-promoting bacterium. AbfB [BAD_1524 gene product; (Lagaert et al. 2010)] belonging to GH51_2 subfamily, and AXH-d3 [BAD_0301 gene product; (van den Broek et al. 2005; Van Laere et al. 1997)] classified into GH43_10 subfamily exhibit AXH-d3 specificity. In contrast, AbfA/AXH-m2,3 [BAD_0423 gene product; (Lagaert et al. 2010; Van Laere et al. 1999)] grouped to GH43_12 subfamily is a typical AXH-m enzyme. In terms of substrate and positional specificity, the BAD_1527 gene product resembles the latter enzyme. A reason for the multiplicity of α-L-arabinofuranosidases showing AXH-m specificity is not clear, although their dissemination within the genome strongly suggests a location in different polysaccharide utilization loci, which are likely to differ in their expression.

The enzyme encoded by the BAD_1527 gene (suggested here to be renamed to *Ba*Abf43C) is classified into the GH43_22 subfamily (http://www.cazy.org/GH43_22.html). So far, four representatives of this subfamily have been characterized in terms of substrate specificity. The GH43_22 domain (AbfA) of *Ruminiclostridium josui* arabinanase Abn43A was found to be an arabinan-specific α-L-arabinofuranosidase not active on rye arabinoxylan. Although a low exo-1,5-α-L-arabinofuranosidase activity was demonstrated on both linear arabinan and AOS, debranching of singly 3-*O*-decorated main chain arabinose residue was the enzyme main activity (Sakka et al. 2019).

The other three characterized GH43_22 representatives are the proteins encoded by the genes found within a single gene cluster of *Bifidobacterium longum* subsp. *longum*. Although the gene cluster encodes also α-L-arabinofuranosidases from other GH43 subfamilies, the three GH43_22 enzymes differ in substrate specificity. The primary target of *Bl*ArafA (a product of BLLJ_1854 gene) seems to be type II arabinogalactan (e.g., from radish root and larch) and sugar beet arabinan. Accordingly, the enzyme released arabinose from internally 3-*O*-arabinosylated β-1,6-galactotriose and methyl 1,2-, 1,5- and mainly 1,3-α-L-arabinofuranobiosides. Heavily substituted gum arabic arabinogalactan served as significantly worse substrate, while wheat arabinoxylan was almost not hydrolyzed (Fujita et al. 2019). *Bl*ArafB (a product of BLLJ_1853 gene) is specific 1,5-α-L-arabinofuranosidase liberating arabinose from sugar beet arabinan but not from wheat arabinoxylan, gum arabic and larch arabinogalactans (Sasaki et al. 2022). Similarly, methyl α-1,5-L-arabinobioside served as a substrate, in contrast to α-1,2- and α-1,3-isomers (Komeno et al. 2019). *Bl*AbfE (a product of BLLJ_1850 gene) and specifically its GH43_22 domain liberated arabinose, which was 1,3-linked to side chain galactose, readily from gum arabic arabinogalactan but hardly from larch arabinogalactan having less complicated side chains (Sasaki et al. 2022). If the same galactose is substituted at position 4 by an additional arabinose, this 3,4-diarabinosylated epitope seems to be a better substrate. However, later it was found that wheat arabinoxylan is at least as good polysaccharide substrate as larch arabinogalactan, in contrast to sugar beet arabinan that was not hydrolyzed. Moreover, the GH43_22 domain of *Bl*ArafE released arabinose from 3-*O*-singly decorated xylose residues, extremely slowly from 2-*O*-singly and not at all from 2,3-diarabinosylated xylose residues (Komeno et al. 2022). Therefore, *Ba*Abf43C studied in this work is most similar to *Bl*ArafE. On arabinoxylan and the oligosaccharides derived thereof both behave as AXH-m enzymes. This specificity is exhibited also on arabinan in case of *Ba*Abf43C, while *Bl*ArafE is inactive, thus *Ba*Abf43C resembles rather *Rj*AbfA when considering action on arabinan. The other difference between *Ba*Abf43C and *Bl*ArafE is their capacity to debranch arabinogalactan, which served as a substrate of *Bl*ArafE only.

We may conclude that the gene BAD_1527 encodes a protein belonging to GH43_22 subfamily ascribed earlier as a β-xylosidase, although it is actually an α-L-arabinofuranosidase suggested to be named *Ba*Abf43C since it represents the third characterized α-L-arabinofuranosidase of *B.* *adolescentis* belonging to GH43 family. *Ba*Abf43C behaves as a typical AXH-m enzyme, releasing arabinose side residues at positions 3 and 2 from singly decorated xylose and arabinose residues of arabinoxylan and arabinan. The enzyme is not active on 2,3-doubly arabinosylated residues, linear xylooligosaccharides, glucuronoxylan, larchwood arabinogalactan and linear arabinan, although short linear arabinooligosaccharides are also slowly hydrolyzed. By its substrate specificity *Ba*Abf43C differs from other GH43_22 members characterized so far. From a physiological point of view, the *Ba*Abf43C widens a spectrum of *B.* *adolescentis* hydrolytic enzymes employed by the probiotic bacterium for the utilization of arabinose-containing plant polysaccharides, in particular arabinoxylan and arabinan, and the corresponding prebiotic oligosaccharides.

### Supplementary Information

Supplementary Material 1

## Data Availability

The authors declare that the data supporting the findings of this study are available within the paper. Should any raw data files be needed in another format they are available from the corresponding author upon reasonable request.

## Abbreviations

AG: arabinogalactan
AOS: α-L-arabinooligosaccharide(s)
AA^3^A: 3^2^-α-L-arabinofuranosyl-arabinotriose
AA^2^^,3^A: 2^2^,3^2^-di-α-L-arabinofuranosyl-arabinotriose
AAA^3^A: 3^2^-α-L-arabinofuranosyl-arabinotetraose
Ara*f*: α-L-arabinofuranosyl
AX: arabinoxylan
AXH: arabinoxylan arabinofuranohydrolase
AXOS: arabinoxylooligosaccharide(s)
A^3^X: 3^2^-α-L-arabinofuranosyl-xylobiose
A^2^XX: 2^3^-α-L-arabinofuranosyl-xylotriose
XA^2^XX: 2^3^-α-L-arabinofuranosyl-xylotetraose
XA^3^XX: 3^3^-α-L-arabinofuranosyl-xylotetraose
A^2^^,3^XX: 2^3^,3^3^-di-α-L-arabinofuranosyl-xylotriose
XA^2^^,3^XX: 2^3^,3^3^-di-α-L-arabinofuranosyl-xylotetraose
CAZy: Carbohydrate Active enZyme
DA: debranched arabinan
GH: glycoside hydrolase
GX: glucuronoxylan
NPA: 4-nitrophenyl α-L-arabinofuranoside
NPX: 4-nitrophenyl β-D-xylopyranoside
RAX: rye arabinoxylan
SBA: sugar beet arabinan
WAX: wheat arabinoxylan
XOS: xylooligosaccharide(s)
Xyl*p*: β-D-xylopyranosyl
