# Supplementary material for: The Bifidobacterium adolescentis BAD_1527 gene encodes GH43_22 α-L-arabinofuranosidase of AXH-m type
Source: AMB Express. 2024 Jul 20;14:83. doi: 10.1186/s13568-024-01738-9 (PMC11264647; doi:10.1186/s13568-024-01738-9)
Supplement: Supplementary file 1 — Supplementary Material 1 [file 13568_2024_1738_MOESM1_ESM.pdf]

AMB Express

Supplementary Material

Original Article

The *Bifidobacterium adolescentis* BAD_1527 gene encodes GH43_22 α-L-arabinofuranosidase of AXH-m type

Walid Fathallah[a,b], Vladimír Puchart[a,*]

Institute of Chemistry, Slovak Academy of Sciences, Dúbravská cesta 9, SK-845 38 Bratislava, Slovakia

* Correspondence: vladimir.puchart@savba.sk

Analysis of 3-D model of *Ba*Abf43C

First we aimed in construction of a model of *Ba*Abf43C studied in our manuscript (having Uniprot accession code A1A3M5). Fortunately, we found out that such a model has already been deposited in AlphaFold database at the web-page

https://alphafold.ebi.ac.uk/entry/A1A3M5

It is the fourth version of the model and it exhibits very high value of confidence, for a vast majority of amino acid residues being greater than 90 %. Besides a few amino acids at N- and C-termini only some surface located loops and two helices are less confident having values between 70 and 90 %.

The model has been subsequently subjected to a search for similar structures deposited in PDB. 192 records were found in total. The similar structures of the GH43 family representatives are listed in Table ST1, where they are sorted by structural similarity score.

Supplementary Table ST1. List of the GH43 enzymes having solved 3-D structures that showed similarity with *Ba*Abf43C model.

PDB Id	Enzyme	Additional hits of the same enzyme	Carbohydrate ligand(s)	GH43 subfamily
4MLG	Uncultured organism β-xylosidase RS223-BX		None	1
3QEF	*Cellvibrio japonicus* arabinan-specific α-1,2-L-arabinofuranosidase *Cj*Abf43A (CJA_3018)	3QED, 3QEE	AA^3A	29
5M8B	*Levilactobacillus brevis* α-L-arabinofuranosidase II *Lb*Araf43 (N624_1993)		None	26
5A8C	*Acetivibrio thermocellus* α-L-arabinofuranosidase *Ct*Abf43A (Cthe_1271)		None	16
5FLW	*Bifidobacterium bifidum* exo-β-1,3-galactanase (BBIF_0009)		None	24
4QQS	*Halothermothrix orenii* α-L-arabinofuranosidase *Ho*Araf43 (Hore_20580)		None	33
5GLR	uncultured bacterium β-xylosidase/α-L-arabinofuranosidase *Co*Xyl43	5GLK, 5GLL, 5GLM, 5GLN, 5GLO, 5GLP, 5GLQ	Xyl_3 + Ara*f*	1
5M8E	*Weissella* sp. 142 α-L-arabinofuranosidase *W*Araf43		None	26
4NOV	*Butyrivibrio proteoclasticus* α-L-arabinofuranosidase Xsa43E (bpr_I2319)		None	29
5C0P	*Bacteroides thetaiotaomicron* endo-arabinanase (BT1873)		None	17

3K1U	*Clostridium acetobutylicum* protein Abf2 (CAC1529)		None	26
1GYH	*Cellvibrio japonicus* endo-α-1,5-L-arabinanase / exo-α-1,5-L-arabinanase *Cj*Arb43A (CJA_0805)		None	5
3KST	*Bacteroides thetaiotaomicron* protein (BT2895)		None	31
1UV4	*Bacillus subtilis* endo-α-1,5-L-arabinanase *Bs*Abn43A (BSU28810)		None	5

The *Ba*Abf43C model was superimposed with all these GH43 structures, particularly those complexed with a carbohydrate ligand, aiming in the identification of amino acids of *Ba*Abf43C involved in the substrate recognition. We focused on the PDB records 5GLR (a complex of GH43_1 *Co*Xyl43 β-xylosidase from an uncultured bacterium with xylotriose and arabinose) and 3QEF (a complex of GH43_29 arabinan-specific α-1,2-L-arabinofuranosidase *Cj*Abf43A from *Cellvibrio japonicus* with AA^3A), see Supplementary Figure S2. The latter seemed to be promising, since it has occupied +1 subsite with the arabinooligosaccharide and -1 subsite harbours ethylene glycol molecule mimicking a carbohydrate. Similarly, in the 5GLR complex, xylotriose is bound to a broader active site and arabinose occupies the enzyme -1 subsite, interacting with catalytically important amino acids.

All the GH43 proteins listed in Table ST1 were included in multiple sequence alignment, along with characterized GH43_22 α-L-arabinofuranosidases (*Rj*Abn43A, *Bl*Abf43A, *Bl*Abf43B and *Bl*Abf43E), *Ba*Abf43C and other characterized *B. adolescentis* α-L-arabinofuranosidases (*Ba*AbfA, *Ba*AbfB and *Ba*AXH-d3). The alignment is shown and discussed in the maintext.

References:

Pettersen EF, Goddard TD, Huang CC, Couch GS, Greenblatt DM, Meng EC, Ferrin TE (2004) UCSF Chimera – a visualization system for exploratory research and analysis. J Comput Chem 25:1605-1612. https://doi.org/10.1002/jcc.20084

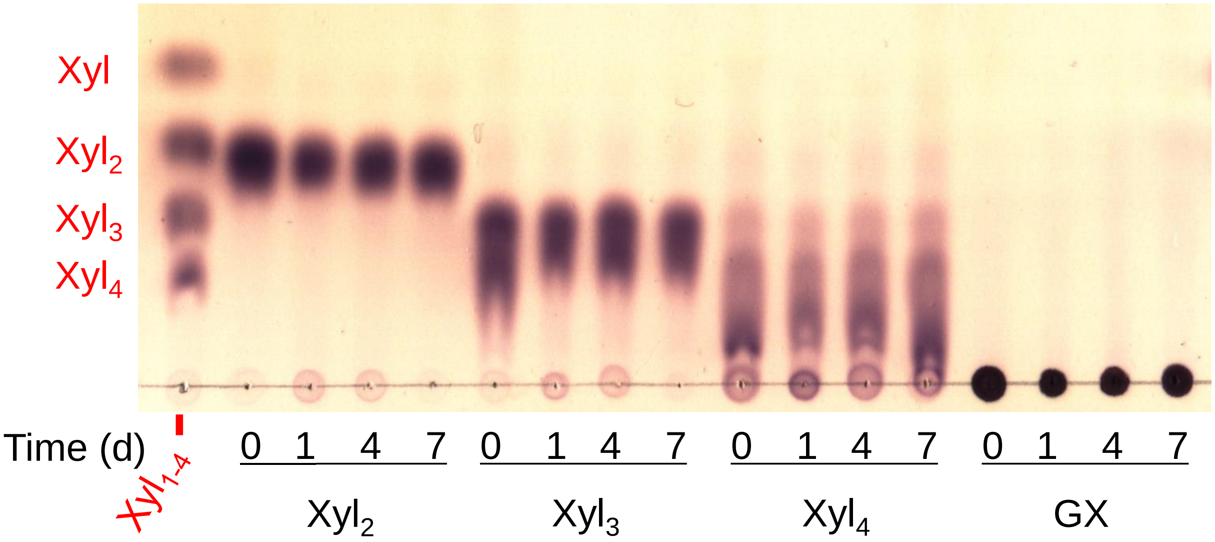

Supplementary Fig. S1. TLC analysis of the hydrolyzates of linear xylooligosaccharides (xylobiose, xylotriose, xylotetraose; 0.1%) and polymeric glucuronoxylan (GX; 1%) by *Ba*Abf43C (0.01 mg/mL). The hydrolysis was performed in 50 mM sodium phosphate buffer, pH 6.0, at 35 °C. The plate was developed in the solvent system of 1-butanol/ethanol/water (10:8:5, by vol.), and the sugars were visualized by orcinol detection reagent. S – xylooligosaccharide standards of xylose to β-1,4-xylotetraose.

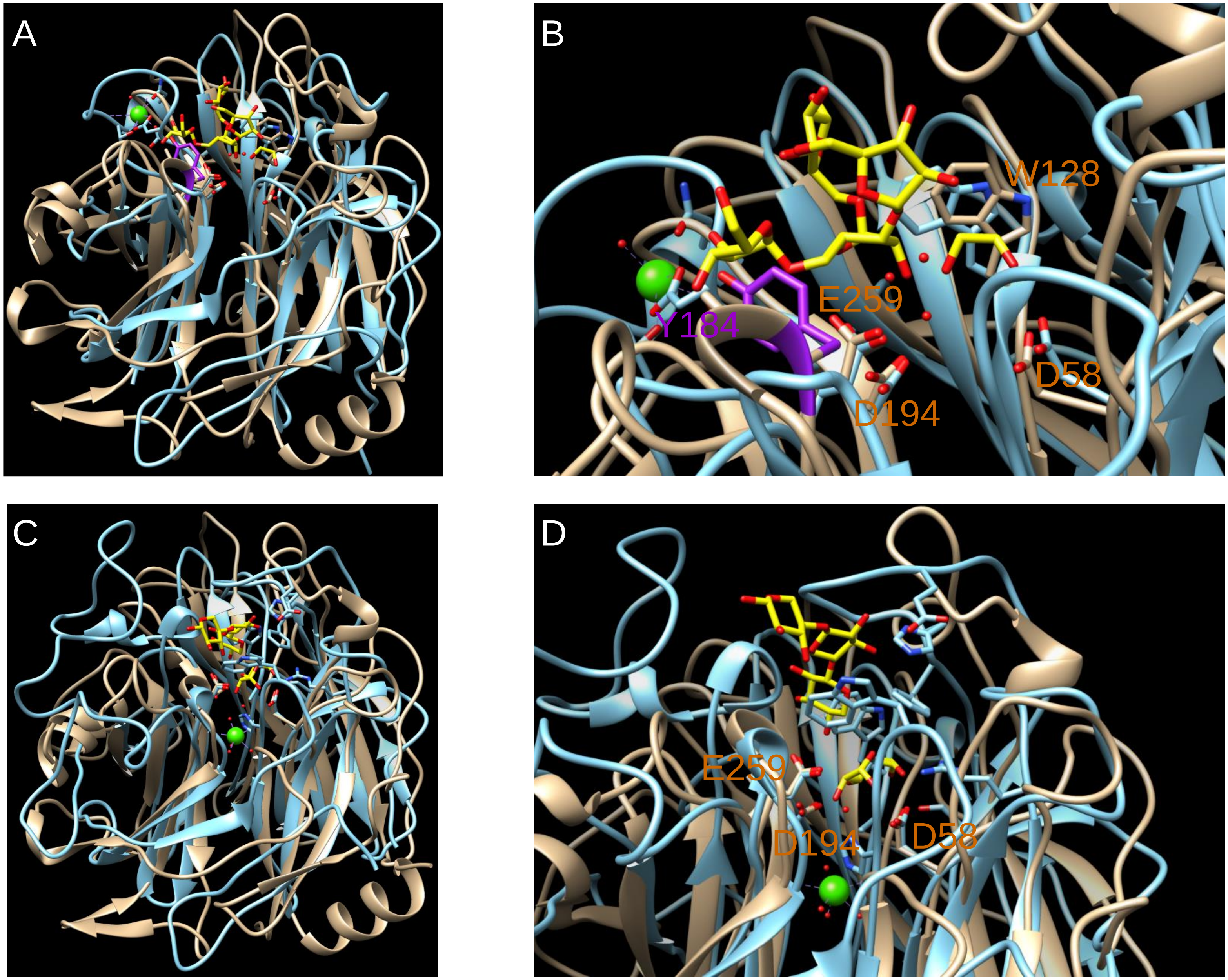
A
B
W128
E259
Y184
D58
D194
C
D
E259
D194
D58

Supplementary Fig. S2. Comparison of 3-D structure of *Ba*Abf43C model with *Cj*Abf43A complex with AA^3A (3QEF; panels A and B), and with *Co*Xyl43 ternary complex with xylotriose and arabinose (5GLR; panels C and D). The *Ba*Abf43C model is shown in light brown colour, while the experimentally determined structures are cyan coloured. Carbon atoms of the ligands are depicted in yellow. Panel A, superposition of entire protein of the model with *Cj*Abf43A. Panel B, detailed view of *Cj*Abf43A active site. A carbohydrate-mimicking ligand, ethylene glycol, is harboured in subsite -1, while the ligand AA^3A occupies the nearby active site subsites. Predicted catalytic residues (D58, D194 and E259) of *Ba*Abf43C as well as the conserved W128 residue are depicted. Side chain of *Ba*Abf43C Y184 (violet) would clash with the arabinooligosaccharide ligand. Panel C, superposition of entire protein of the model with *Co*Xyl43. Panel D, detailed view of *Co*Xyl43 active site. The ligand Xyl_3 occupies positive binding subsites. Another carbohydrate- ligand, arabinose, is harboured in subsite -1. The picture was prepared using Chimera (Pettersen et al. 2004)
